# Footprints of Fascination: Digital Traces of Public Engagement with Particle Physics on CERN's Social Media Platforms

**DOI:** 10.1371/journal.pone.0156409

**Published:** 2016-05-27

**Authors:** Kate Kahle, Aviv J. Sharon, Ayelet Baram-Tsabari

**Affiliations:** 1 Education, Communication and Outreach Group, Editorial Content Development Section, CERN, Geneva, Switzerland; 2 Faculty of Education in Science and Technology, Technion–Israel Institute of Technology, Haifa, Israel; Universidad Veracruzana, MEXICO

## Abstract

Although the scientific community increasingly recognizes that its communication with the public may shape civic engagement with science, few studies have characterized how this communication occurs online. Social media plays a growing role in this engagement, yet it is not known if or how different platforms support different types of engagement. This study sets out to explore how users engage with science communication items on different platforms of social media, and what are the characteristics of the items that tend to attract large numbers of user interactions. Here, user interactions with almost identical items on five of CERN's social media platforms were quantitatively compared over an eight-week period, including likes, comments, shares, click-throughs, and time spent on CERN's site. The most popular items were qualitatively analyzed for content features. Findings indicate that as audience size of a social media platform grows, the total rate of engagement with content tends to grow as well. However, per user, engagement tends to decline with audience size. Across all platforms, similar topics tend to consistently receive high engagement. In particular, awe-inspiring imagery tends to frequently attract high engagement across platforms, independent of newsworthiness. To our knowledge, this study provides the first cross-platform characterization of public engagement with science on social media. Findings, although focused on particle physics, have a multidisciplinary nature; they may serve to benchmark social media analytics for assessing science communication activities in various domains. Evidence-based suggestions for practitioners are also offered.

## Introduction

Members of the public might need to know about science for a variety of reasons and purposes. These range from the mundane, such as making everyday personal consumer and health decisions, to the more sophisticated, such as participating in decisions on socio-scientific topics and appreciating science as a part of human culture [1]. Promoting mutual understanding between the scientific community and the public is also important for maintaining legitimacy and funding for science itself [1,2].

Social media, such as *Facebook* and *Twitter*, may facilitate direct communication between experts and the public more than traditional media has enabled in the past. They allow both for scholar participation in wider discussions and communication with new audiences, and for public engagement with scientific research and participation in the social context in which it takes place [3–5]. However, while people are increasingly spending time consuming, generating and exchanging content on social media [6], only few studies have characterized how the public engages with *scientific* information on these media [3]. Specifically, little is known on how different types of content and different social media platforms shape different types of public engagement with science online. To characterize these effects, this study makes use of digital trace data of public engagement with science. These data go far beyond what could be gathered from engagement with traditional media such as surveys and audience measurement tools for radio and television. We explore user interactions with almost identical content items cross-posted on five social media platforms of the European Organization for Nuclear Research (CERN).

## Literature Review

### Public Engagement with Science Online

Much of the public's engagement with science takes place online. According to a survey conducted in the US in 2014, the Internet was the public's primary source for science and technology information (42%, up from 35% in 2010) [7]. Similarly, in a 2014 survey, 67% of US respondents said that the Internet was their primary source of *specific* information about scientific issues, up from 63% in 2012 [8,9]. Increasingly, US lay audiences are relying on non-journalistic online sources, such as blogs and social media platforms, as sources of information about science [10].

Relatively little is known about user engagement with scientific information online [10]. Some of the existing research on this topic has focused specifically on characterizing (1) *seeking*, (2) *commenting* on and (3) *sharing* scientific information in specific contexts over the Internet.

#### Information-seeking

Observational findings suggest that educational activities and media attention to scientific issues motivate people to seek scientific information. Thus, for example, search volumes for queries such as "Swine Flu Vaccine", "West Nile Virus" and "Global Warming" tend to be associated with media coverage, whereas search volumes for queries such as "Biology", "Chemistry" and "DNA" tend to be associated with the academic calendar [11]. Searches for scientific topics featured in the news, such as science-related Nobel prizes, grow quickly (1.2–1.3% per minute in the first 9–10 hours after the announcement), but this attention tends to be short-lived, and declines by half within a week [12]. Additionally, experimental findings suggest that people tend to search for an emerging technology more often if they support it, or if they anticipate that they will have to discuss it with people who hold views on it that differ from their own [13].

#### Commenting

Comments can reveal what meanings are derived by readers from coverage of science-related topics, and what resources they bring to the dialogue between science and society [14]. Thus, commenting can be considered an enactment of scientific literacy, visible to other people in the community [15].

Research conducted on user comments on *news websites* has indicated that comments correlate moderately positively with clicks (*r* = 0.71) and that there are differences in the topics that tend to receive clicks and those that tend to receive comments: The former topics tend to arouse curiosity, whereas the latter tend to create controversy [16]. Comment sections foster discourse on science-related issues, such as animal experimentation, in unexpected directions, introduced by the user community. Arguments raised in these discussions may go against scientific consensus. User-introduced topics were also more fruitful than topics featured in the articles, in terms of the number of comments [14]. In comment sections of stories about the scientific evidence for breastfeeding, the implications of findings on daily life are critiqued through the lens of personal experience [17]. Exposure to online, user-to-user uncivil comments about emerging technologies, such as nanotechnology, can affect how readers perceive the risks of these technologies [18].

Whereas the comment sections on news articles and blog posts are originally intended to allow responses to topics raised by the editors of the hosting websites, any commenter can raise an issue on *online discussion forums*. These forums are increasingly being used to discuss science-related topics, such as headlice eradication [19] and infant weaning [20] among parents. On these forums, personal experience of parents may be placed on a par with, and sometimes regarded more highly than, formal scientific knowledge [19]. Participants may also use forums to critique the quality of media coverage of scientific research [20].

#### Sharing

Like commenting, sharing is a visible action that users can take, which may foster science-related online discussion. Studies conducted on observational data from the *New York Times* website and from an experimental setting have indicated that users tend to share science-related content that is surprising, interesting, otherwise entertaining, positive, useful, or inspiring awe, anger or anxiety. Users are motivated to share such content for a variety of reasons, including: (1) to make themselves "look good" in the eyes of others, and (2) to enhance their social bonds with others [21,22]. Sharing is the behaviour underlying virality, a phenomenon which has increased in speed, reach and frequency. Viral information flow is unique in that a specific piece of information is forwarded (or "shared") by many people to their social networks over a short period of time, and then that message spreads to different, often distant networks. This pattern has the power to capture public attention, and subsequently transform attitudes and change courses of action [23]. Inducing this type of information flow could be considered the holy grail of science communicators. Some examples of popular and perhaps viral science videos on *YouTube* include a demonstration of what happens to a cheeseburger dipped in concentrated hydrochloric acid [24] and a discussion of whether it is better to walk or run in the rain, if one wishes to remain as dry as possible [25].

These studies have contributed to our understanding of engagement with science on news websites and search engines, and to understanding behaviours such as clicking, sharing and commenting. However, relatively little attention has been paid, so far, to the characterization of *other* types of user engagement with online scientific information, such as "liking". Also, little is known about the relationships between clicking, commenting and other behaviours, such as sharing and spending time on a science-related website.

### Social Media and Public Engagement with Science

Social media such as *Facebook* and *Twitter* are "digital Web 2.0 platforms that facilitate information-sharing, user-created content and collaboration across people" [26]. On these platforms, users can typically create a (semi-)public profile, create connections with others and view and correspond with people they are connected to. These platforms have become popular worldwide. In fact, *Facebook* reports that in December 2015, 1.04 billion unique users actively used the platform on an average day [27]. Moreover, for many people, especially in industrialized societies, social media have become an integral part of daily life [28]. These prevalent yet poorly understood platforms take many forms, creating different contexts that differently shape user cognition, affect and behaviour. Most of the relevant research focuses on *specific* platforms, precluding a broader understanding of social media and of the ways people use them [26].

Like social media research in general, literature on public engagement with science on social media is relatively unsystematic, and each study generally focuses on a specific platform. For example, a study on engagement with *YouTube* videos about organ donations found that the vast majority of user comments on such videos were positive [29]. Another study focused on engagement with messages provided by U.S. health agencies on *Twitter*, and found that tweets about activities and behaviours, chemicals, drugs, and disorders tend to be retweeted more often than tweets on other topics [30]. Yet another *Twitter* study found that information about a new influenza vaccine tended to flow via retweets between users who shared the same sentiments about it [31]. Lastly, another study described public engagement with a marine research institute on *Facebook*, finding that posting video and images increased the reach of posts, and so did posting long stories. However, the researchers also found that interactions with the audience did not extend beyond a few exchanges of questions and answers between users and the page administrator [32].

To conclude, researchers are only beginning to understand the effects of different social media platforms on engagement with science, and have yet to develop rigorous methodologies for this purpose. Here, we provide a quantitative and qualitative characterization of public engagement with particle physics across five social media platforms and describe a method to conduct such cross-platform comparisons.

Two research questions guided this study:

1. How do users engage with scientific information on different social media platforms, when controlling for content?
2. What are the characteristics of the most popular scientific information items on social media in terms of user interactions?

## Research Field

To address these research questions, authentic social media analytics data from the European Organization for Nuclear Research (CERN) were analyzed. CERN is an international scientific research organization founded in 1954 as one of Europe's first joint ventures, and now has 21 member states. The CERN laboratory sits astride the Franco-Swiss border near Geneva, where researchers study particle physics using the world's largest and most complex scientific instruments. Notably, CERN is also the birthplace of the World Wide Web.

Particle physics provides an especially challenging topic for science communication, since it is "segregated from society on many counts" [33]. It is abstract, esoteric, and uniquely awe-inspiring, yet it is dependent on massive publicly funded machines "beyond the budget of any single research organization, or indeed, any single country" [34]. Qualitative research suggests that the members of the particle physics community are firmly committed to the international, supracultural image of science [35]. With international funding and global science comes a need to communicate with the worldwide public.

Social media is one of CERN’s tools to communicate with the public, as part of a broader communication strategy [36]. The organization is currently active on *Twitter*, *Facebook*, *YouTube*, *Google+*, *Instagram* and *LinkedIn*.

CERN began using *Twitter* in 2008 and by August 2014 had more than a million followers on this platform. During the 4 July 2012 Higgs boson announcement, CERN’s live tweets reached journalists faster than the press release, and helped contribute to worldwide coverage of the particle discovery. *Twiplomacy* reports from 2013 and 2015 ranked CERN as one of the most effective international organizations on Twitter [37,38].

### CERN's Social Media Strategy

There are three main goals to CERN's social media strategy:

1. To begin a journey: Key messages are disseminated by repackaging CERN’s online content for the different social media channels. Most social media content contains links back to the CERN website, potentially starting a journey to find out more.
2. To foster engagement: CERN’s presence on social media channels is aimed to foster engagement in the general public and to help form an online community of stakeholders interested in the laboratory and its work.
3. To retain positive sentiment: Social media is a way to reach the general public and a way to monitor sentiment towards the organization. CERN aspires to retain a strong brand identity by keeping sentiments toward it positive and handling negative sentiments constructively by responding as appropriate to questions or concerns.

These goals can be linked to particular approaches to public engagement with science: educational, engagement and marketing, respectively. While education and marketing can both be considered associated with the deficit model in science communication, engagement can be related to the dialogical model [39], as outlined in Table 1. User behaviours on social media can be used as key performance indicators (KPI)–measurable values that organizations use to benchmark their effectiveness in achieving objectives.

**Table 1:** Mapping CERN's social media strategy to approaches to science communication and key performance indicators.

	CERN's Goals	Approaches to Science Communication	Key Performance Indicators (KPIs)
1.	Begin a journey	Educational	Click-throughs; average visit time, retention rate
2.	Foster engagement	Engagement	Shares; Comments
3.	Retain positive sentiment	Marketing	Likes

By analysing user behaviours in detail, in particular when social media posts were well-received, an organization can evaluate the effectiveness of social media content and whether it is in line with its strategic objectives, thus helping to shape future content.

### CERN's Social Media Platforms

This study focused on a sub-set of five of CERN’s social media platforms: Two *Twitter* accounts (in English and in French); *Facebook*, *Google+* and *Instagram (LinkedIn* and *YouTube* were not included because the regularity and content of posts on these platforms does not match the others). Each platform differs in audience numbers and demographics, detailed in Table 2.

**Table 2:** Demographics of the Audience on Each Platform. Data recorded during the data collection period 17 October– 11 December 2014.

Platform	*Facebook*	*Twitter* English	*Twitter* French	*Google+*	*Instagram*
**Audience at start** of data collection	343K	1.03M	12.2K	104K	100
**Audience at end** of data collection	367K	1.06M	12.6K	110K	1.19K
**Gender F% / M%**	31.3 / 68.7	29.9 / 70.1	41.5 / 58.5	29 / 71	37.9 / 62.1
**Age 13–17** (F% / M%) ^1^	0.9 / 3	0.8 / 2.8	1.3 / 2	1 / 2.9	1.6 / 2.8
**Age 18–24** (F% / M%) ^1^	3.7 / 11	4 / 11	5.7 / 8.3	4.8 / 10.7	5.9 / 11.8
**Age 25–34** (F% / M%) ^1^	9.1 / 21.1	7.7 / 21.5	10.6 / 17.8	8.2 / 21.3	12.6 / 19.7
**Age 35–44** (F% / M%) ^1^	8.8 / 17.9	9.4 / 17.8	10.5 / 14.6	7.7 / 18.1	10.1 / 14.5
**Age 45–54** (F% / M%) ^1^	6.5 / 11.9	5.9 / 12.7	9.8 / 11.4	5.2 / 13	5.8 / 9.6
**Age 55–64** (F% / M%) ^1^	1.6 / 2.8	1.5 / 3.2	2.7 / 3.2	1.5 / 3.7	1.6 / 2.6
**Age 65+** (F% / M%) ^1^	0.6 / 1.1	0.6 / 1.1	0.9 / 1.2	0.6 / 1.3	0.4 / 1
**Top 5 countries of origin**	USA, India, UK, Italy, Turkey	USA, UK, Italy, Spain, Switzerland	France, Switzerland, Canada, Belgium, USA	Ukraine, USA, India, Denmark, UK	US, Italy, UK, France, Turkey

^1^ of the total percentage of that gender

## Methodology

**Fig 1:**
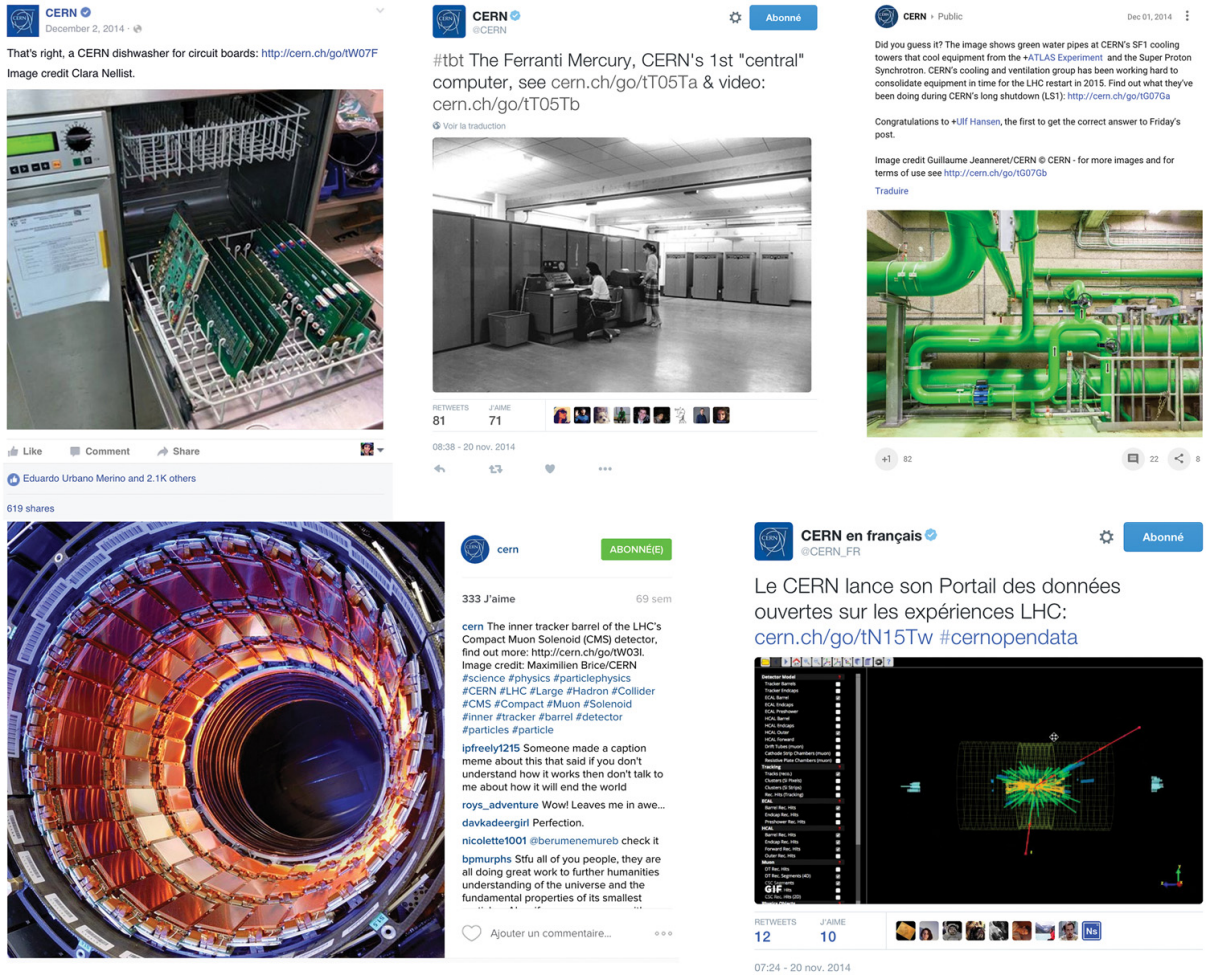
Examples of the five social media platforms and four content types. Top row, left to right: one of the 40 Wow items, shown on *Facebook*; one of the 40 Throwback Thursday items, shown on *Twitter* English; one of the 40 Guess What It Is items, shown on *Google+*. Bottom row, left to right: one of the 40 Wow posts, shown on *Instagram*; one of the 94 news items, shown on *Twitter* French.

Forty-eight (48) different topics, each featuring a unique image (e.g. an illustration or photograph), were (cross-)posted on five of CERN's social media platforms over eight weeks in 2014 (17 October– 11 December). Each topic belonged to one of the following four categories: (1) News, (2) "Guess What It Is" (GWII), which featured mysterious images (e.g. images of unusual scientific instruments), (3) "Throwback Thursday" (TBT), which featured historical images, and (4) "Wow", featuring awe-inspiring images. Items (i.e., *Facebook* statuses, *Twitter* tweets, etc.) relating to each topic were posted on one or more of these platforms. Example items are provided in Fig 1. In most cases, but not all, topics were cross-posted on all five platforms. In total, this yielded 214 items (Table 3). For all platforms, each item contained either one or two links, yielding 225 links in total. Items on *Instagram* had inactive links in the text due to the technical constraints of the *Instagram* platform (n_Instagram_ = 32). To follow a link, a user would have to copy-paste the text into a browser. (Table 4).

**Table 3:** Cross-tabulation of items by social media platform and content type.

Content Type	Platform					Total
	*Facebook*	*Twitter* English	*Twitter* French	*Google+*	*Instagram*	
**News**	24	23	17	22	8	**94**
**GWII**	8	8	8	8	8	**40**
**TBT**	8	8	8	8	8	**40**
**Wow**	8	8	8	8	8	**40**
**Total**	**48**	**47**	**41**	**46**	**32**	**214**

**Table 4:** Cross-tabulation of links by social media platform and content type.

Item Type	Platform					Total
	*Facebook*	*Twitter* English	*Twitter* French	*Google+*	*Instagram*	
**News**	30	29	20	28	0	**107**
**GWII**	12	11	11	12	0	**46**
**TBT**	9	9	9	9	0	**36**
**Wow**	9	9	9	9	0	**36**
**Total**	**60**	**58**	**49**	**58**	**0**	**225**

Users of the respective platforms were either exposed to the items or not exposed to them, depending on their individual usage habits and the technical settings of the particular platform. *Facebook*, for example, uses an algorithm that filters what the audience sees in their news feed, such that "[o]f the 1,500+ stories a person might see whenever they log onto *Facebook*, News Feed displays approximately 300" [40]. The News Feed algorithm uses several factors to determine top stories shared by people and by Pages of businesses, brands or organizations, including the number of comments, who shared the story, and what type of post it is (for example, photo, video, or status update) [41]. CERN relies on organic reach only, meaning that no payment was given to any of the platforms in exchange for increased exposure to the items.

Three user behaviours were recorded for each *item*: (1) "Likes", "Favourites" (*Twitter*) or "+1" (*Google+*) (hereafter "likes"); (2) Comments or replies (hereafter "comments"); and (3) Shares or retweets (hereafter "shares") (Table 5).

**Table 5:** Interactive behaviours recorded on social media in this study.

Platform	Item-Level Behaviours			Link-Level Behaviours		
	1	2	3	4	5	6
	Like	Comment	Share	Click Through	Visit Duration	Retention Rate
***Facebook***	Like	Comment	Share	Click Through	Visit Duration	Retention Rate
***Twitter***	Favourite	Reply	Retweet	Click Through	Visit Duration	Retention Rate
***Google+***	+1	Comment	Share	Click Through	Visit Duration	Retention Rate
***Instagram***	❤	Comment	n/a	n/a	n/a	n/a

Different platforms use different names for similar behaviours. This study uses the *Facebook* terminology.

In addition, three user behaviours were recorded for each *link*: (4) Click-throughs–The number of times the link was clicked; (5) The average visit duration on CERN's page if the link was clicked; (6) The retention rate–The percent of visitors who clicked on the link and then clicked on other links within the page. The first four user behaviours occur on the social media platform, whereas the last two relate to on-site behaviours. Because of technical constraints of the *Instagram* platform, only the first two behaviours (1–2) were recorded for that platform (n_Instagram_ = 32) (Table 5).

### Data Collection

User behaviours were recorded using *Engagor* (http://www.engagor.com), which records likes, shares and comments. CERN's “shortened URL service” recorded click-throughs, visit durations and retention rates. Data collection period spanned 17 October– 11 December 2014. Engagement is typically in the first 24 hours after a post is published. With CERN’s global audience, to take into account time-zones and subsequent shares of content, it was decided to collect the data for each post approximately one week after the post was published.

### Statistical Analysis

Raw data was normalized by audience size of the platform on the date of item posting, and standard z-scores were computed. For instance, if an item on *Facebook* received one standard deviation more comments (per 1,000 followers) than the mean for comments on *Facebook* items, its "comments" z-score was 1. Items with at least one user behaviour statistic scoring |z| ≥ 1.96 were considered "high-engagement" items (if z ≥ +1.96) or "low-engagement" items (if z ≤ −1.96). Assuming a normal distribution of user engagement, these thresholds would yield the top and bottom 2.5% of observations. Since the resulting distributions was far from normal (see "Results"), no low-engagement items were found using this method. Results for each user behaviour were analysed separately using a series of univariate two-way ANOVA tests, followed by Scheffé post-hoc tests when significant *F* values were found.

## Results

### Audience Sizes and Engagement Rates

An average post on CERN’s social media platforms received 161.68 likes (SD 358.8), 9.5 comments (SD 31.93), 64.37 shares (SD 143.8), and 93 click-throughs (SD 166.1). For users who clicked on the links in the posts, the mean visit duration on the web pages that the links led to was 16.27 seconds (SD 34.67) and the mean retention rate was 5.45% (SD 8.2%). Averages for each user behaviour and platform are detailed in Table 6.

**Table 6:** User interactions per item with CERN items on different social media platforms, by platform.

Platform	Statistics	Likes	Comments	Shares	Click-Throughs	Avg. Visit Duration (s)	Retention Rate (%)
***Facebook***	**Mean**	433.15	27.85	66.77	91.45	14.62	4.683
	**N**	48	48	48	60	60	60
	**SD**	674.54	63.23	116	186.62	44.19	6.53
***Twitter* English**	**Mean**	122.21	7.8	159.98	224.28	9.28	3.57
	**N**	47	47	47	58	58	58
	**SD**	134.07	10.56	228.95	209.44	9.32	1.94
***Twitter* French**	**Mean**	2.54	0.22	5.83	11.49	34.63	10.92
	**N**	41	41	41	49	49	49
	**SD**	2.47	.65	5.29	8.5	46.42	12.97
***Google+***	**Mean**	95.24	4.72	16.35	32.52	9.45	3.5
	**N**	46	46	46	58	58	58
	**SD**	71.61	5.9	21.7	38.56	20.31	6.37
***Instagram***	**Mean**	111.84	3.25	-	-	-	-
	**N**	32	32	-	-	-	-
	**SD**	39.48	3.49	-	-	-	-
**Total**	**Mean**	**161.68**	**9.5**	**64.37**	**93.08**	**16.27**	**5.45**
	**N**	**214**	**214**	**182**	**225**	**225**	**225**
	**SD**	**358.81**	**31.93**	**143.79**	**166.11**	**34.67**	**8.2**

On average, the most common behaviours found were *Facebook* likes (433.15 Interactions per Item (IPI), SD 674.54), *Twitter* English favourites (122.21 IPI, SD 134.07) and *Instagram* likes (111.84 IPI, SD 39.47). On *Twitter* English, shares and click-throughs were also common with 159.98 shares per item (SD 228.95) and 224.27 click-throughs per link (SD 209.44).

Notably, the coefficients of variation of these behaviours per item were extremely high, at approximately 2.2. The large variation in each of these measures limits how much meaning can be derived from the overall averages.

**Fig 2:**
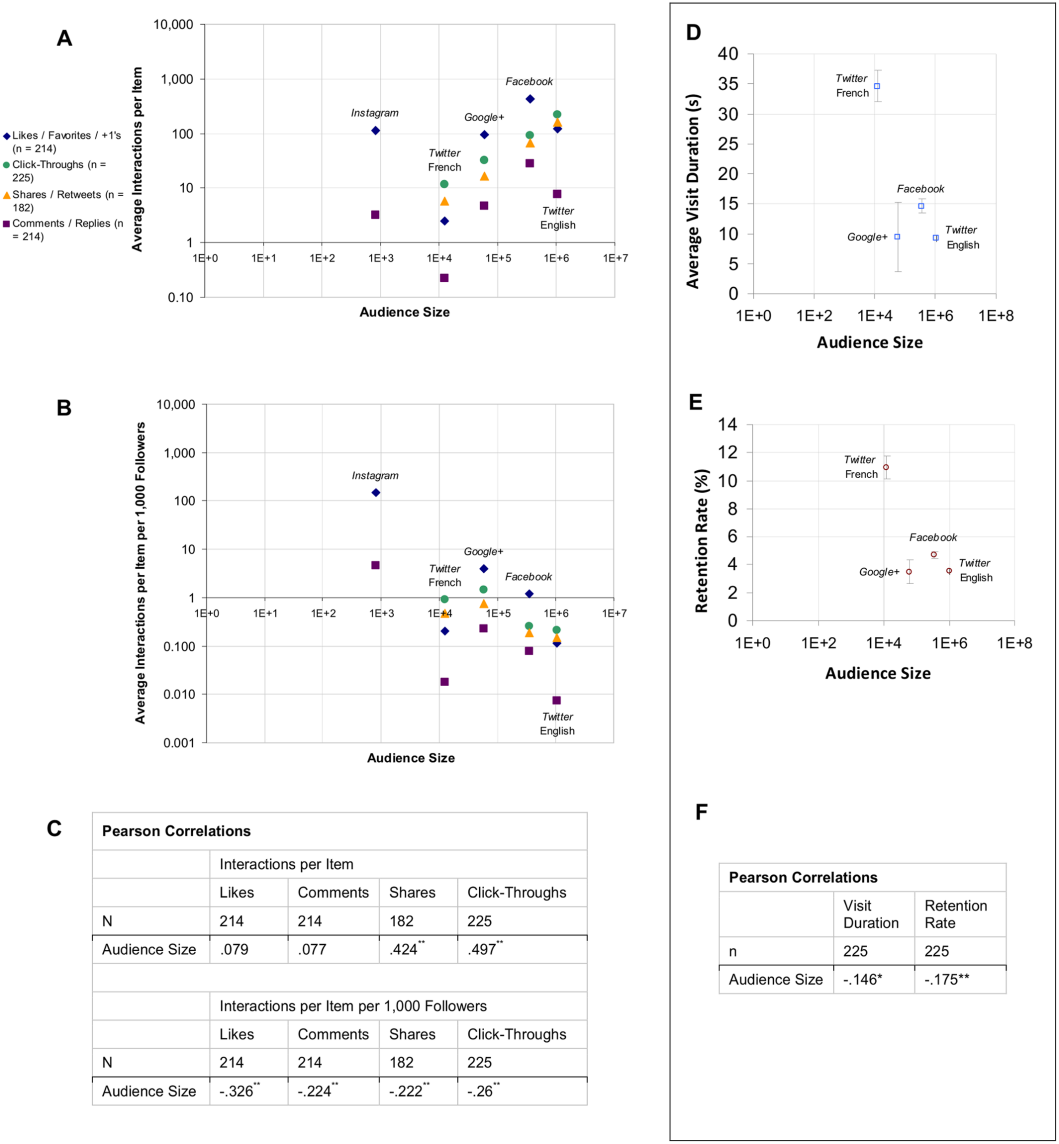
Average rates of user interactions with items posted on CERN's social media platforms. (A) User interaction rates without control for audience size. (B) User interaction rates with control for audience size. (C) Pearson correlations between audience size and user interactions relating to behaviours on the social media platform. (D) Visit durations of visitors arriving by links posted on different platforms, by audience sizes of the platforms. (E) Average retention rates of visitors arriving by links posted on different platforms, by audience sizes of the platforms. (F) Pearson correlations between audience size and user interactions relating to on-site behaviour. *. Correlation is significant at the 0.05 level (2-tailed). **. Correlation is significant at the 0.01 level (2-tailed). Error bars denote the standard error of the mean.

Part of this large variation is explained by audience size. Namely, items on platforms with larger numbers of followers tended to receive more interactive behaviours overall (Fig 2A). After controlling for audience size, however, user interactions were found to be most common on *Instagram* and *Google+*. Some of the most common behaviours found in the study were *Instagram* likes (149 Interactions per Item per 1,000 Users (IPI/kU), SD 54.66), *Instagram* comments (4.62 IPI/kU, SD 5.41), *Google+* "+1"s (4.02 IPI/kU, SD 5.54) and *Google+* click-throughs (1.47 IPI/kU, SD 2.98) (Fig 2B). Visit durations and retention rates were highest for *Twitter* French, with users spending 34.63 seconds on average (SD 46.42), and 10.92% (SD 12.97%) clicking on on-site links, respectively.

Audience size correlated significantly and moderately positively with total shares and click-throughs, and weakly negatively for visit duration (r = -0.146, *p* < 0.01) and retention rate (r = -0.175, *p* < 0.01). No significant correlation was found between audience size and total likes or comments. However, when controlling for audience size, likes, comments, shares and click-throughs occurred less often as audience sizes grew. Correlations were moderately negative except for the correlation between audience size and likes, which was strongly negative (Fig 2C). In summary, larger audiences correlated with higher engagement rates in total; However, per-user, engagement declined with audience size.

Having said that, audience size is not everything. The educational goals of visit durations and retention rates (Table 1, row 1) were similarly attained among *Twitter* English, *Facebook* and *Google+* although they have very different audience sizes, while users arriving to the URLs through *Twitter* French stay on the page for a much longer time, by a factor of 2 to 3 (Fig 2D and 2E). This may be a result of the language itself: to convey the same information takes, in general, more words in French than in English [42]. In English, 140 characters can be enough to convey a message. In French, this is not always the case, resulting in enigmatic text that encourages the reader to click through to find out more. Once on the webpage, more French text is used to convey the message than the equivalent English so naturally the time spent on the page increases. If the web page content is tailored to the audience e.g. a full publication in French, the audience is more likely to click further to read additional content.

### Platforms, Item Types, and the Interactions Between Them

Overall, the most popular behaviour on different item types was likes on "Wow" items (285.93 IPI, SD 703.74). "Wow" items also received relatively many click-throughs (142.56 IPI, SD 249.65) and shares (115.88 IPI, SD 244.12). Other notable behaviours include likes on "News" items (163.57 IPI, SD 264.41), click-throughs on "News" items" (98.36 IPI, SD 166.58), and likes on GWII items (104.55 IPI, SD 92.01) (Table 7).

**Table 7:** User interactions per item with CERN items on different social media platforms, by item type.

Item Type	Likes	Comments	Shares	Click-Throughs	Avg. Visit Duration (s)	Retention Rate (%)
**GWII**						
Mean	104.55	5.53	36.13	71.35	23.94	7.00
N	40	40	32	46	46	46
SD	92.01	6.39	51.27	103.99	42.86	11.98
**News**						
Mean	163.57	10.48	71.04	98.36	13.80	5.84
N	94	94	86	107	107	107
SD	264.41	24.11	138.18	166.58	19.28	7.88
**TBT**						
Mean	90.1	3.5	23.19	55.72	18.03	3.61
N	40	40	32	36	36	36
SD	90.90	4.63	26.70	107.98	60.23	5.47
**Wow**						
Mean	285.93	17.20	115.88	142.56	12.03	4.14
N	40	40	32	36	36	36
SD	703.74	63.27	244.12	249.65	21.47	4.31
**Total**						
**Mean**	**161.68**	**9.50**	**64.37**	**93.08**	**16.27**	**5.45**
**N**	**214**	**214**	**182**	**225**	**225**	**225**
**SD**	**358.81**	**31.93**	**143.79**	**166.11**	**34.67**	**8.20**

A series of ANOVA tests revealed different combined effects of social media platforms and item types on different user behaviours.

#### Likes

**Fig 3:**
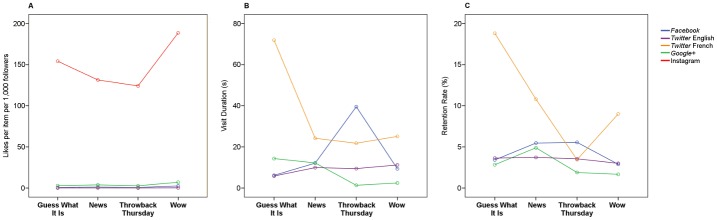
Interactions between likes, visit durations and retention rates, by platform and item type. (A) Likes per item per 1,000 followers, by platform and item type. (B) Visit durations (C) Retention rates, by platform and item type. Y-axes show estimated marginal means, which reflect main effects, while controlling for other effects. GWII: Guess What It Is. TBT: Throwback Thursday.

There was a significant interaction between platform and item type on the number of likes per user (*F*_(12,194)_ = 3.46, *p* < 0.001). Especially, it seems that the combined effect of Wow images and the *Instagram* platform yields many more likes than any other combination of platform and item type (Fig 3A).

#### Visit duration and retention rate

There was a significant interaction between platform and item type on the average visit duration (per user) (*F*_(9, 209)_ = 2.629, *p* < 0.01) and on retention rate (*F*_(9, 209)_ = 2.075, *p* < 0.05). Among users who clicked on links, *Twitter* French users uniquely tended to spend much more time on pages that Guess What It Is links led to than any other user on any other platform or item type (Fig 3B). This interaction is also reflected in retention rate data (Fig 3C).

#### Comments

In the case of comments, platform has a significant effect on user behaviour, but item type does not. For example, platform was found to have a significant effect on the number of comments (per user) (*F*_(4,12)_ = 31.684, *p* < 0.001). Post-hoc tests revealed that *Instagram* had significantly more comments (per user) than any other platform (*p* < 0.001). However, no significant effect of item type on comments (per user) was found, nor was a significant interaction of platform and item type found.

#### Click-throughs

Similar to commenting, platform was found to have a significant effect on clicking on links (*F*_(3, 209)_ = 6.956, *p* < 0.001). Post-hoc tests revealed that on average, links on *Google+* received more click-throughs (per user) than links on *Facebook* or *Twitter* (*p* < 0.05). However, no significant effect of item type on click-throughs (per user) was found, nor of the interaction between platform and item type.

#### Sharing

Last but not least, sharing was found to be a unique behaviour in this study, in that no significant effects of item type or platform on shares (per user) were found.

### Characterizing High Engagement

#### Fluctuation over time

**Fig 4:**
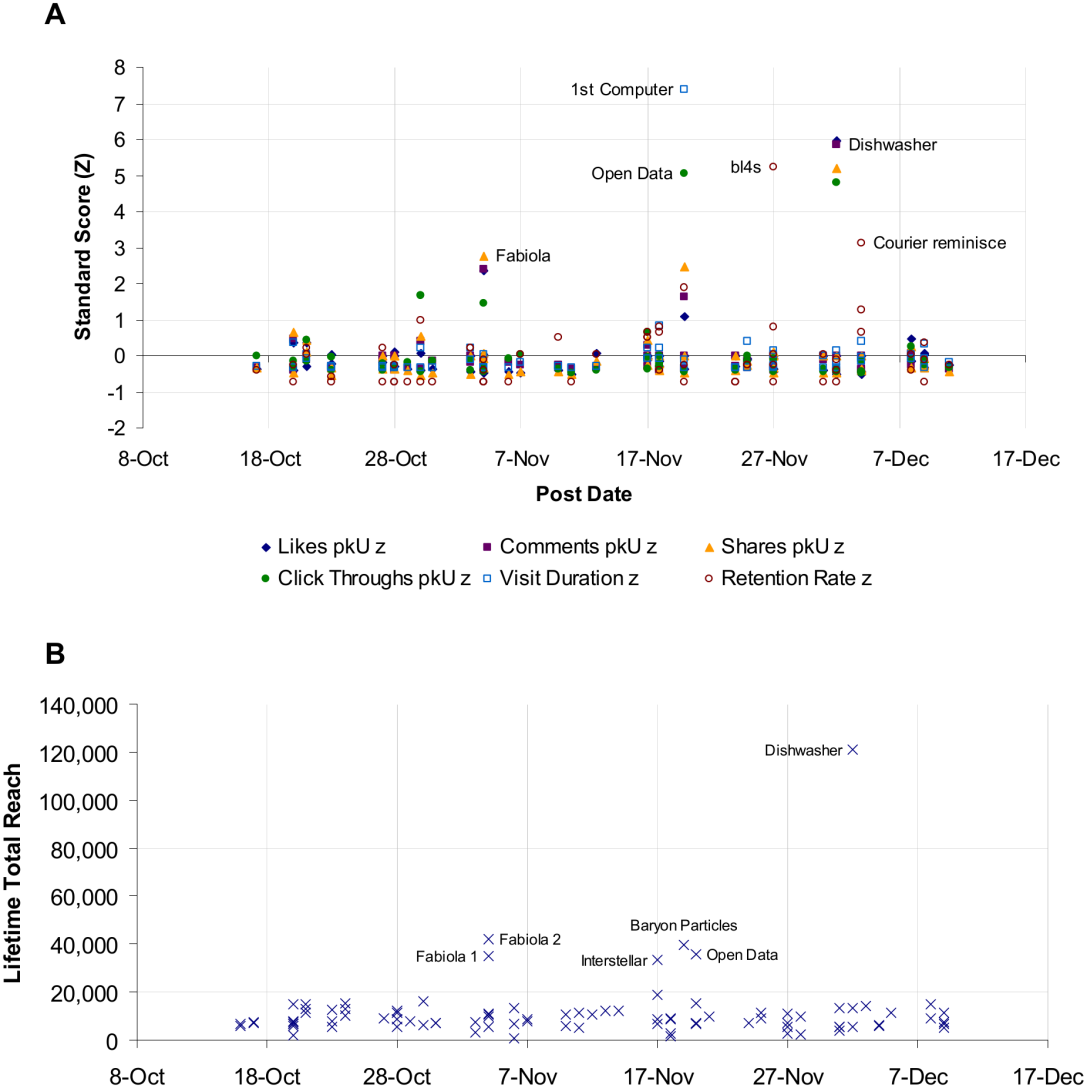
User engagement with scientific content and reach on CERN's *Facebook* page over time, October–December 2014. (A) User engagement with scientific items over time. Zero represents the mean rate for each user behaviour on *Facebook* per item per 1,000 *Facebook* followers on the day of sampling: Likes 1.21 IPI/kU (SD 1.86); Comments 0.0779 IPI/kU (SD 0.17), Shares 0.187 IPI/kU (SD 0.32); Click-throughs 0.256 IPI/kU (SD 0.52). pkU: Per Thousand Users. Z: Z-score. The size of CERN's *Facebook* audience size grew from 343,000 to 367,000 over the course of the study. (B) Reach of scientific items over time. Reach is the total number of *Facebook* users the item was served to.

User engagement with items on CERN’s social media platforms fluctuated strongly over time. Fig 4 represents the pattern of user interactions with items posted on CERN's *Facebook* page over the time period studied, normalized by daily audience size. In total, the audience typically engaged with items at a constant rate of interactions throughout the study, as illustrated by the cluster of observations near the x axis. In addition, several outliers were found, some with z-scores as high as 5 or more, meaning that for certain items, user behaviours occurred as often as 5 standard deviations more than the means for those behaviours on the respective platforms. A similar pattern of user behaviour was found in most platforms studied.

#### High-engagement items

Thirty-five (35) high-engagement items were found in the study, comprising more than 16% of the 214 items included in the sample. These were defined as items with at least one user behaviour statistic scoring *z* ≥ 1.96. As an example, the six high-engagement items for CERN's *Facebook* page are labelled in Fig 4A. The point labelled "Open Data" in Fig 4A, for instance, refers to click-throughs on a link in the *Facebook* announcement that CERN had launched an Open Data Portal to make the data of LHC experiments publicly available. This item received high standard scores on *Facebook* in terms of click-throughs per thousand users (*z* = 5.05) and shares per thousand users (*z* = 2.47). Hence, it was considered a high engagement item posted on the *Facebook* platform. (Since the distribution was strongly right-skewed, no low-engagement items were identified in the study.) The lifetime total reach of these *Facebook* posts, a measure indicating the number of users who potentially could interact with the post, was similar at 11,490 users (SD 13,900). Most of the posts reached around 10,000 users (Mean 10,300, SD 7,565) except for one outlier, "Dishwasher," which concerned a dishwasher for circuit boards, and reached over 121,000 users (Fig 4B). This indicates that in not all cases was user engagement necessarily driven by increased reach.

#### Associations between high engagement items and topics

High engagement across platforms is significantly associated with item topic. Six (6) topics were repeatedly popular across multiple platforms (hereafter "recurring" high-engagement topics), representing 19 items of the 35 "high engagement" items. For example, the "Open Data" topic received high engagement scores not only on *Facebook* but also on *Google+*, *Twitter* English and *Twitter* French, making it a recurring high-engagement topic. By contrast, another 16 high-engagement items each represented 16 different topics that received high engagement in just one platform (hereafter: "unique" high-engagement items). These data indicate an association between high engagement and item topic (χ^2^_(47)_ = 80.054, n = 214, *p* < 0.01, Cramer's *V* = 0.612).

Some characteristics of the high-engagement topics are that they may have referred to (1) news items receiving attention from traditional media (e.g. the "Fabiola" topic), or (2) a surprising or awe-inspiring image (e.g. "CMS", "Dishwasher" and "Pipes") (Table 8).

**Table 8:** Recurring high engagement topics.

	Recurring High Engagement Topic Code	Type	Image Caption	Recurred as High Engagement Item on…
1.	Fabiola	News	"CERN Council selects Italian physicist, Dr Fabiola Gianotti, as CERN’s next Director-General"	*Facebook*, *Twitter* English, *Twitter* French
2.	Open Data	News	"CERN launches Open Data Portal to make public the data of LHC experiments"	*Facebook*, *Google+*, *Twitter* English, *Twitter* French
3.	Pipes	Guess What It Is	"CERN's cooling & ventilation systems get refreshed"	*Google+*, *Twitter* French
4.	1^st^ Computer	Throwback Thursday	"The Ferranti Mercury, CERN's 1st 'central' computer"	*Facebook*, *Twitter* English
5.	CMS	Wow	"The LHC’s Compact Muon Solenoid (CMS) detector"	*Instagram*, *Twitter* English, *Twitter* French
6.	Dishwasher	Wow	"That's right, a CERN dishwasher for circuit boards"	*Facebook*, *Google+*, *Instagram*, *Twitter* English, *Twitter* French

### Research Limitations

The main methodological limitation in this study stems from the architecture of the platforms. The items posted were not necessarily seen by all CERN’s subscribers. The "organic reach" is determined by the technical settings of the platforms, and may be affected by many different variables. For example, one study found that organic reach increases on a given *Facebook* item if another item was posted on the platform the day before [30].

The results are based on data collected from October to December 2014, however changes may have occurred since then at multiple levels: from the CERN social media strategy and behaviour, to the architecture of the platforms, as well as the audiences, their preferences and the general online communication landscape. Concerning CERN’s strategy and posting behaviour, this has remained consistent with the data-taking period. However, platform architectures are regularly changed and updated. Since our findings indicate that the platform itself influences user behaviour, it follows that changes in the platform may have an effect. For example, *Twitter* has implemented a new feed algorithm [43]. *Google+* has been fully redesigned [44]. *Facebook* have not only changed the way that content from pages are delivered to the audience [45], they are also placing more and more emphasis on video content, particularly live or immersive videos, over other types of content [46]. One recent *Facebook* update now allows people to express their feelings as “reactions” to the information published [47]. These changes call for more elaborate future research in this topic, with fine-tuned analysis that looks at both the comments and the reaction icons. The online communication landscape in general has become more mobile, with some audiences shifting to other social media platforms such as *Snapchat*. Notwithstanding the dynamics of this field, our systematic study still provides valuable benchmarks for science communication on the platforms within the study as well as for future platforms.

Also, the items do not represent a randomly distributed, year-round sample. "Throwback Thursday" items, for example, were posted only on Thursdays, "Guess What It Is" items were posted only on Mondays, and "Wow" items were posted only on Tuesdays. The 8 weeks included in the sample were not randomly distributed but represent only the end of the calendar year. These temporal characteristics of the sample add further possible confounding factors to the study.

Next, while the number of users for some platforms was high, two platforms had relatively few followers during the time of the study: *Twitter* French and *Instagram*. Hence, when outliers appeared on these platforms, it could be a result of chance. Additionally, when focusing on visit duration and retention rate, often the numbers that clicked-through in the first place were so small as to be only the hardened fans determined to spend time to find out more. Future work could include setting thresholds for inclusion in datasets, or devising measures that integrate numbers of users who clicked through and the time they spent on site.

Finally, another limitation stems from the reliance on datasets provided by for-profit corporations such as *Engagor*. *Engagor* does not divulge the algorithms used to generate the data. Future work could focus on developing open, free tools for generating social media analytics for research purposes.

## Evidence-Based Insights for Practice

How does our research inform practice? Specifically the practice of using social media for science communication. Here, we provide some evidence-based insights with examples (Table 9).

**Table 9:** Content characteristics and related user behaviour on social media.

	Likes	Comments	Shares	Click-throughs	Visit duration	Retention rate
News	✓	✓	✓	✓		
Image	✓	✓	✓	✓	✓	✓
Animation	✓		✓			
Video/Virtual tour on webpage					✓	✓
Discussion		✓				
Clickbait				✓		
Tailored content					✓	✓
Human story						✓

### News

With the increased prevalence of social media, news stories from an organisation are no longer confined to traditional media. Many people, especially younger generations, get their news directly from social media [48], either receiving it directly from the organisation or via a share from their social network. With such a wealth of news on social media, audiences react by liking, commenting, sharing and clicking-through, but stay very little time on the webpage, quickly consuming the content and moving on. This type of post is therefore more focused on marketing and engagement and less on education in terms of the social media strategy.

### Images and Animation

Users respond more readily to images than text on social media [49,50]. In order to control for this variable, all posts in the study contained an image. Furthermore, *Facebook* algorithms are configured to promote image-based posts to a wider audience. In this study, animations were also used on *Twitter* and *Google+*, receiving a relatively strong reaction in terms of likes and shares [51,52]. Of the 35 high engagement items, more than half were not news related but involved beautiful images (e.g. [53]) or surprising images (e.g. [54]). Meaning that an organisation can use imagery on social media for all three strategic themes: marketing, engagement and education. That said, educational psychology research suggests that illustrations and entertaining text may result in less retention of important information. It suggests that cognitive interest and emotional interest may be at conflict with regard to educational purposes, such as learning scientific explanations [55].

### Video or Virtual Tour

This study found that visit duration on the webpage linked from social media increased when a video or virtual tour was embedded in the page (e.g. [56]), especially when the video was placed further down the page and seen after reading the text (e.g. [57]). Retention rate increased when viewers were led to YouTube videos (e.g. [58,59]) or playlists (e.g. [60,61]) as many users clicked to watch additional videos.

### Comments and Discussion

Similarly to what was found in [14], comments foster discourse sometimes in unexpected directions (e.g. [62]). Arguments growing from user-introduced topics sometimes dominated the discussion and increased the number of comments unrelated to the posts themselves (e.g. [63]). The organisation has created a space for more engagement, but has less control of the message. What the organisation can control is how the comments are addressed, either with a response or by enforcing a comment policy [64]. Thus, where comments are concerned, it is important to read carefully for content and tone to really understand the effectiveness of a post.

### Clickbait

When a social media post had a strange image and enigmatic text, readers were intrigued to find out more and lured by the “clickbait”–online content whose main purpose is to attract attention and encourage visitors to click on a link to a particular web page. When comparing a *Twitter* post in English with its French equivalent (S1 and S2 Figs), the English-language followers had all the information they needed in the tweet so were less motivated to click-through than the French. Whereas the French tweet had 2.5 times the average click-throughs.

### Tailored Content

Science communication needs to always have the audience in mind, so tailoring content to a given audience is best practise. Take, for example, the visually minded audience of *Instagram*. A beautiful ALICE detector image [62] had greater than average likes and comments, showing marketing and engagement strategies were fulfilled. For education, the linked webpage also needs to be tailored to the audience. CERN’s *Google+* audience enjoy solving weekly quizzes so spent longer on a webpage to solve strange captionless images [65]. *Twitter* French users who clicked on links leading to a whole publication of content in their native language (e.g. [66]) spent longer on the site and clicked through multiple stories.

### Human Story

A human focus is one of the most established attention-grabbing features in media, and this is no different for a science-minded audience. One of the biggest stories during the data taking period was that Fabiola Gianotti would be the next Director General of CERN. The comments discussed different aspects of her identity as a scientist, an Italian and a female, relating to her and congratulating her on a personal level. In another example, a *Facebook* post took people to a series of stories written in the first person [67]. Although the click-throughs were not higher than average, those that did click were engaged in the history and kept clicking further to read the full long-form article.

## Concluding Discussion

How does the public engage with science communication items on different platforms of social media? To some extent, engagement is similar irrespective of platform, but in some respects it differs. The small *Instagram* audience at the time of the study was quick to click on the "Like" and "Comment" buttons, especially on awe-inspiring images. *Twitter* French and *Google+* users tend to click on links more often, and the *Twitter* French users who click on links spend a long time reading the CERN webpage. However, across all platforms, user engagement with scientific items on social media tends to fluctuate due to frequent “high engagement” items. Often (but not always) the same “high engagement” topics attract interactions across platforms. Awe-inspiring imagery, for example, is especially likely to lead to high engagement (similar to findings described in [21]).

Some of these differences can perhaps be explained by four factors: (1) Platform effects; (2) Audience effects; and (3) Content effects.

### Platform Effects

The ways digital tools such as social media platforms are designed may affect what we can and would want to use them for. By using them, "we implicitly accept the rules designed into the tools by the organizations that created them" [23]. For example, by algorithmically predetermining which *Facebook* users might see an item, the potential audience for engagement with that item is limited *a priori*. Moreover, *Instagram* does not have a share button and does not allow clickable links in image captions. Consequently, different social media platforms might promote different types of engagement with science, and shape different kinds of learning outcomes.

### Audience Effects

On average, smaller audiences, such as *Instagram* in CERN's case, seem to be more engaged audiences. Perhaps in new accounts, "early adopters" might tend to be more engaged users. Also, large audiences might tend to include many inactive followers. Some "high engagement" topics may be specific to certain platforms because of the unique characteristics of the audiences on different platforms (e.g., the opening of a film in French-speaking Switzerland and in France was of particular interest to followers on *Twitter* French).

### Content Effects

Scientific items on social media that tend to attract large numbers of user interactions include some awe-inspiring imagery as well as news items that were newsworthy enough to receive attention from the traditional mainstream media.

Particle physics requires large investments of public money, and has stood under public scrutiny over safety concerns over black holes [68], but otherwise it seems to be regarded with a positive sentiment, and it does not seem to be as controversial a topic as other sciences. This may explain why commenting was found to be such a relatively rare behaviour in this study compared with other behaviours, across all platforms [16]. We predict that hot-button topics such as animal research or genetically modified foods are likely to attract more comments under similar conditions [14].

In addition, other communication strategies for particle physics may result in different engagement behaviour, for example, Symmetry Magazine [69], a magazine of particle physics published jointly by Fermilab and SLAC laboratories, adopts a fun, accessible voice in its communications. Further research would be needed to determine how the different approaches influence user behaviour. Also, CERN did not always respond to comments, due to limited resources. This is now changing with more CERN scientists getting involved in discussions and may affect future commenting behaviour.

Although quantitative KPI benchmarks were not set, the high-engagement items that exceed the average user behaviours can be used to evaluate which goals (e.g., marketing, education, or engagement) were achieved, and to compare which goals were achieved more effectively than the others. Among the 35 high engagement items, the most common behaviours implicating high engagement were visit duration for 14 items (relating to the educational goal), comments for 13 items (engagement), likes for 11 items (marketing) and shares for 10 items (engagement). Note that often high engagement items had multiple behaviours associated with them. This study demonstrates how platform and content effects affect user behaviours, and offers predictions for future practice with regard to different goals.

Indicators such as *Facebook* organic reach may serve as a proxy for viral reach, as shown in previous work [32]. The combined effects of platform characteristics and content characteristics on virality could serve as a topic for further research, describing characteristics associated with virality of scientific content.

To our knowledge, this study provides the first quantitative description of public engagement with science on social media, across several platforms. It extends findings developed in other contexts, such as news websites and surveys. The ecological validity of the study derives from the fact that it analyses digital traces of the spontaneous reactions of authentic users (rather than of a sample of undergraduate students) specifically on real items (rather than on contrived items) on real social media platforms (rather than in a mock social media platform designed for experimental purposes). Findings may serve for benchmarking social media analytics for science communication activities in the future. In turn, this study may inform the design of science communication campaigns that serve audiences' informational needs and interests, and may contribute to audience members' lifelong learning of science.

## Supporting Information

S1 Fig Tweet about TOTEM detectors, posted on *Twitter* English. (JPG)

S2 Fig Corresponding tweet about TOTEM detectors, posted on *Twitter* French. The English tweet had an average number of click-throughs. In contrast, the equivalent French tweet had 2.5 times the average click-throughs. The French text was more enigmatic and said less than the English, encouraging readers to click to find out more.
(JPG)

S1 File Digital Traces of Public Engagement with Particle Physics on CERN's Social Media Platforms. (XLS)

S2 File *Facebook* lifetime total reach statistics. (XLS)
