# Supplementary figures and images for: Footprints of Fascination: Digital Traces of Public Engagement with Particle Physics on CERN's Social Media Platforms
Source: PLoS One. 2016 May 27;11(5):e0156409. doi: 10.1371/journal.pone.0156409 (PMC4883777; doi:10.1371/journal.pone.0156409)

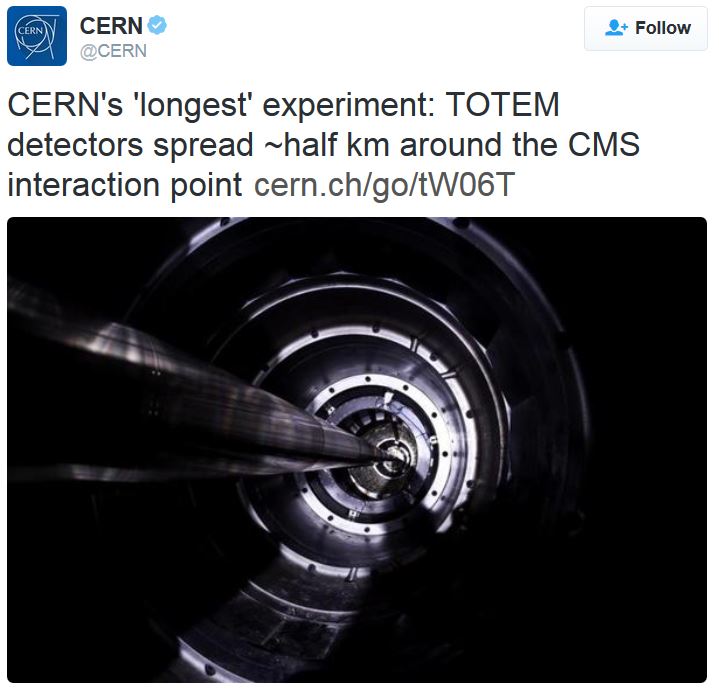

Supplement: S1 Fig — (JPG) [file pone.0156409.s001.JPG]

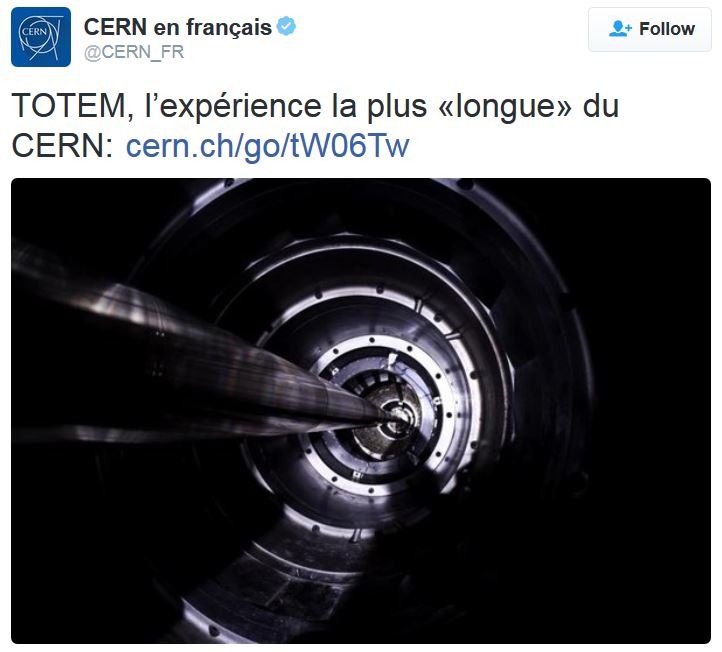

Supplement: S2 Fig — The English tweet had an average number of click-throughs. In contrast, the equivalent French tweet had 2.5 times the average click-throughs. The French text was more enigmatic and said less than the English, encouraging readers to click to find out more. (JPG) [file pone.0156409.s002.JPG]
